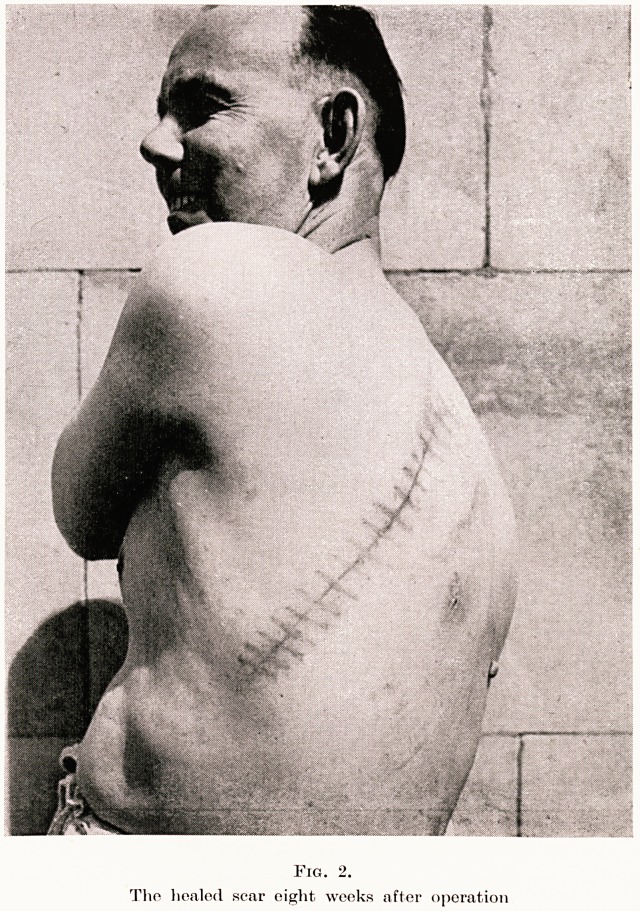# A Case of Lobectomy for Bronchiectasis
*The patient was shown at a meeting of the Bristol Medico-Chirurgical Society on Wednesday, 12th January, 1938.


**Published:** 1938

**Authors:** A. Wilfrid Adams

**Affiliations:** Senior Assistant Surgeon, Bristol Royal Infirmary; Surgeon, Bristol Children's Hospital


### A CASE OF LOBECTOMY FOR BRONCHIECTASIS.*

BY

A. Wilfrid Adams, M.S., F.R.C.S.,
*Senior Assistant Surgeon, Bristol Royal Infirmary ;*
*Surgeon, Bristol Children's Hospital.*

---

This communication is made as the surgery of lobectomy is in its infancy and the success in this patient is encouraging.

The case was particularly unfavourable owing to the age of the patient, over forty years, the rigid chest wall, and the complete obliteration of the pleural space by adhesions. The prolonged operation involved did not prevent a successful issue. Corroboration of the importance of these factors is afforded by the reports of cases operated on by Tudor Edwards.[1]

*History.*—The symptoms in Mr. J. F., aged forty-two years, asserted themselves with alarming vigour first on 15th May, 1935, by an hour's expectoration of foul pus and blood. He collapsed that evening, and the trouble kept him in bed for eight weeks. There had been mild pain over the lower left ribs for eighteen months previously, but only on exertion.

The putrid expectoration lasted a day or two, recurred fortnightly, and was pronounced by the doctor

* The patient was shown at a meeting of the Bristol Medico-Chirurgical Society on Wednesday, 12th January, 1938.

as typical of bronchiectasis. It was accompanied by attacks of hæmoptysis, and on one occasion he was nearly choked by a pint of blood, pus and membranous substance. Permanent pain developed in the lower left chest, often like a "hot coal." He became very dyspnœic, and finally, for two months, had morning vomiting.

Examination in December, 1936, showed a man of stocky build and ruddy complexion. The pulse was 108. There was diminished movement and impaired percussion note at the left base. A tender liver edge was palpable. Plain skiagram showed opacity of the left lower lobe, suggesting bronchiectasis. The sputum was repeatedly negative for tubercle bacilli.

After a few months postural drainage his expectoration diminished and became negligible, and the bronchogram (Fig. 1) clearly revealed left lower lobe bronchiectasis. Preoperative artificial pneumothorax was attempted, but failed owing to adherent pleura.

16th June, 1937. *Operation.* Lower lobectomy of left lung under spinal anæsthesia. (Percaine).

The seventh left intercostal space was slit and 1 in. of rib above resected near its neck. The lung was densely adherent except over the pericardium. Difficulty in defining the diseased base of the lung from the parietes was most daunting, especially in his big rigid chest. But the feeling that his only chance was "now or never" prevented me from relinquishing the effort to complete the operation in one stage. Although the semi-necrotic tissue tore, no visible pus exuded from the lung. The pedicle was first commanded by a tourniquet, then divided, and the bronchial and vascular openings closed by mattress

### PLATE VII.

Fig. 1.

Bronchiectasis in lower left lobe demonstrated by intratracheal injection of lipiodol.

### PLATE VIII.

Fig. 2.

The healed scar eight weeks after operation sutures. The stump was oversewn. During this period blood transfusion commenced. A No. 26 Malecot catheter drain was inserted through the ninth interspace and the chest wall closed. He was rather grey and poorly even before the operation was commenced, and the pulse was very feeble during the procedure. It improved at the close and there was no coldness or sweating.

The lobe had been converted into airless, pulpy, friable tissue surrounding the dilated bronchi. Dr. Fraser reported some small areas of tuberculosis in the specimen.

He appreciated the oxygen tent to which he was returned, distress was never apparent, nor did it ensue when the tent was removed at sixty hours. He never seemed breathless and only coughed up a few blobs of bloody mucus. The airtight drain was joined by a glass connection to a tube ending under water in a bedside bottle. About a pint of bloody fluid collected during the first twenty-four hours, but this rapidly lessened. This underwater drainage ensured the maintenance of negative intrapleural pressure and gave evidence that the catheter was not blocked, as shown by the oscillating column of fluid.

Intermittent pain in the chest was the only symptom that troubled him during convalescence, and by 13th July, 1937, his temperature and pulse were normal. In the skiagrams the upper lung appeared to have expanded to the diaphragm and no pus was aspirable from the chest, so the tube was withdrawn. He expectorated a few blobs of nummular sputum about this time and on 10th August, 1937, left the Infirmary.

The photo (Fig. 2) of the patient on this date is by Mr. Wilfrid Willway, our surgical registrar, who kindly helped with the operation.

In January, 1938, he reported the symptoms had completely left him and his sole complaint was pain deep to the scar, due to adhesions or intercostal neuritis, and not preventing him eating and sleeping well and attending to his duties as a publican. His weight is, he thinks, increased (14 st. 4 lbs.). On auscultation the breath sounds are satisfactory, as is the skiagram.

REFERENCE.

[1] A. Tudor Edwards and G. Price Thomas, *British Journal of Surgery,* vol. xxii, No. 86, October, 1934, p. 310.

## Figures and Tables

**Fig. 1. f1:**
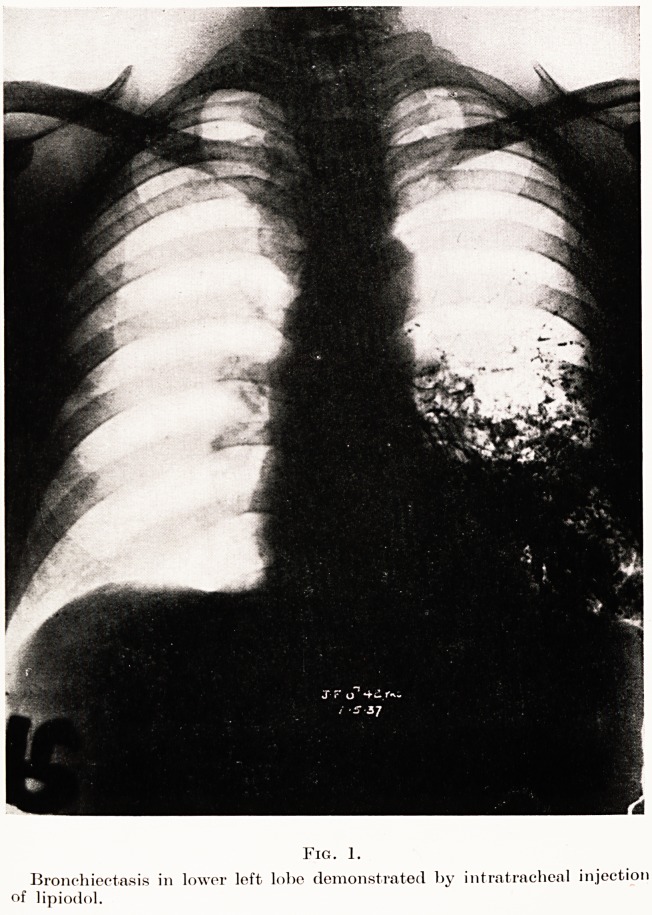


**Fig. 2. f2:**